# Chronic Kidney Disease: Combined Effects of Gene Polymorphisms of Tissue Inhibitors of Metalloproteinase 3, Total Urinary Arsenic, and Blood Lead Concentration

**DOI:** 10.3390/ijerph20031886

**Published:** 2023-01-19

**Authors:** Ying-Chin Lin, Ya-Li Huang, Horng-Sheng Shiue, Sheng-Lun Hsu, Yu-Mei Hsueh

**Affiliations:** 1 Department of Family Medicine, Wan Fang Hospital, Taipei Medical University, Taipei 110, Taiwan; 2 Department of Family Medicine, School of Medicine, College of Medicine, Taipei Medical University, Taipei 110, Taiwan; 3 Department of Occupational Medicine, Wan Fang Hospital, Taipei Medical University, Taipei 110, Taiwan; 4 Department of Public Health, School of Medicine, College of Medicine, Taipei Medical University, Taipei 110, Taiwan; 5 Department of Chinese Medicine, College of Medicine, Chang Gung University, Taoyuan 333, Taiwan

**Keywords:** tissue inhibitor of metalloproteinase 3, polymorphisms, total urinary arsenic, blood lead, chronic kidney disease

## Abstract

The tissue inhibitor of metalloproteinase 3 (TIMP3) is known to be an anti-fibrotic factor. Arsenic, lead, and cadmium exposure and selenium intake may affect TIMP3 expression. The downregulation of TIMP3 expression is related to kidney fibrosis. Genotypes of TIMP3 are related to hypertension and cardiovascular diseases. Therefore, this study explored whether TIMP3 polymorphism is associated with hypertension-related chronic kidney disease (CKD). In addition, the combined effects of TIMP3 polymorphism and total urinary arsenic, blood lead and cadmium, and plasma selenium concentrations on CKD, were investigated. This was a case-control study, with 213 CKD patients and 423 age- and sex-matched controls recruited. Polymerase chain reaction-restriction fragment length polymorphism was used to determine TIMP3 gene polymorphisms. The concentrations of urinary arsenic species, plasma selenium, and blood lead and cadmium were measured. The odds ratio (OR) of CKD in the TIMP3rs9609643 GA/AA genotype was higher than that of the GG genotype at high levels of total urinary arsenic and blood lead; the OR and 95% confidence interval (CI) were 0.57 (0.31–1.05) and 0.52 (0.30–0.93), respectively, after multivariate adjustment. High blood lead levels tended to interact with the TIMP3rs9609643 GG genotype to increase the OR of CKD, and gave the highest OR (95% CI) for CKD of 5.97 (2.60–13.67). Our study supports a possible role for the TIMP3rs9609643 risk genotype combined with high total urinary arsenic or with high blood lead concentration to increase the OR of CKD.

## 1. Introduction

Chronic kidney disease (CKD) affects >10% of the world’s population and has emerged as one of leading causes of mortality worldwide [1]. Using an estimated glomerular filtration rate (eGFR) < 60 mL/min/1.73 m^2^ to define CKD, the prevalence of CKD in Taiwan was 11.9%, of which only 3.5% of patients were aware of their disease [2]. The incidence of end stage renal disease in Taiwan ranks first in the world [3]; therefore, exploring the etiology of CKD is an important issue in Taiwan.

Our recent study found that high plasma selenium concentrations significantly increased eGFR and decreased the odds ratio (OR) for CKD, and blood cadmium and lead concentrations, and total urinary arsenic concentration significantly decreased eGFR and increased OR for CKD [4]. Exposure to arsenic, lead and cadmium can cause tubular degeneration, fibrosis, hemorrhage and vacuolation in rat kidney tissue [5]. Studies have also found that arsenic, lead and cadmium can induce oxidative stress and cause nephrotoxicity [6,7,8]. Selenium can reduce the oxidative stress and fibrosis caused by these metals [5,9]. However, the mechanisms by which blood cadmium and lead, total urinary arsenic, and plasma selenium concentrations are associated with CKD have not been fully elucidated.

The tissue inhibitor of metalloproteinase 3 (*TIMP3*) is a physiological inhibitor of matrix metalloproteinases (MMPs). A disruption of the balance between MMPs and TIMPs can alter the stability and normal function in the extracellular matrix (ECM) and lead to abnormal tissue remodeling and homeostasis [10]. Of the four TIMPs, *TIMP3* is the only one with an affinity for proteoglycans in the ECM [11], and it is also known to have anti-fibrotic effects [12]. The downregulation of *TIMP3* may enhance the extent of tubule interstitial fibrosis (TIF) [12]. Recent studies reported that TIF was associated with CKD development and progression [13].

The increased expression of *TIMP3* was observed in human kidney 2 epithelial cells under arsenic exposure [14]. Exposure to cadmium during pregnancy causes structural changes in fetal kidney tissue, which can be detected by increased levels of some kidney injury biomarkers in amniotic fluid such as albumin, osteopontin, vascular endothelial growth factor and TIMP1 [15]. One study found that with high blood lead concentration, both MMP2 and MMP9 were significantly increased, while TIMP2 was significantly decreased in the placenta of women [16]. A low-selenium diet may lead to decreased selenium content in adult rat kidneys, upregulation of MMP1 and MMP3 and downregulation of their inhibitors (TIMP1 and *TIMP3*), resulting in renal ultrastructural and ECM damage [17]. Exposure to arsenic, lead and cadmium may be positively or negatively associated with *TIMP3*, whereas selenium appears to be positively associated with *TIMP3*. However, results of current research are inconsistent.

The *TIMP3* genes are located on chromosome 22q12.1 [18] and *TIMP3* is a 24-kDa secreted protein that binds strongly to the ECM. A study of Chinese Han people found that *TIMP3*rs9619311 TC+CC or *TIMP3*rs2234921 AG+GG genotypes had a significantly higher risk of carotid plaque than those with the TT or AA genotypes, respectively [19]. A recent study reported that the *TIMP3*rs9619311 TT genotype had a significantly higher risk of essential hypertension than the TC+CC genotype [20]. One study demonstrated that *TIMP3*rs9609643 and *TIMP3*rs8136803 affect individual differences in breast cancer susceptibility and survival [21]. A study found that a significantly higher risk of colorectal cancer for *TIMP3*rs715521 AG+AA than GG genotype [22]. Current studies found that *TIMP3* gene polymorphisms were associated with carotid plaques, hypertension, and cancer. Whether *TIMP3* polymorphisms are associated with hypertension-related CKD remains to be explored. This study explored the association between *TIMP3* genotypes and CKD. In addition, the combined effects of *TIMP3* genotype and arsenic, lead or cadmium body burden and plasma selenium concentrations on CKD were evaluated.

## 2. Materials and Methods

### 2.1. Study Subjects

This was a hospital-based case-control study. From September 2005 to September 2011, 214 CKD patients and 423 age- and sex-matched healthy controls were recruited at Taipei Medical University Hospital and Taipei Wanfang Medical Center [23]. This study was approved by the Institutional Review Board of Taipei Medical University (N202101029). All study subjects were interviewed by questionnaires and biological samples were collected after they provided their informed consent.

Based on blood urea nitrogen, serum creatinine, and proteinuria, the Modification of Diet in Renal Disease formula was used to calculate eGFR by nephrologists from Taipei Medical University Hospital and Taipei Wanfang Hospital to determine the different stages CKD patients: eGFR (mL/min/1.73 m^2^) = 186.3 × serum creatinine^−1.154^ × age^−0.203^ × 1.212 (if patient is black) × 0.742 (if female) [24].

### 2.2. Interview and Bio-Specimen Collection

Study subjects were interviewed using a structured questionnaire by well-trained interviewers. The contents of the questionnaire included sociodemographic data; lifestyle such as cigarette smoking habit and consumption of alcohol, coffee, and tea; analgesic usage; and disease history.

An EDTA-vacuum syringe was used to collect 5–8 mL of blood, and the buffy coats were separated for DNA extraction and analysis of *TIMP3*rs9619311, *TIMP3*rs11547635, *TIMP3*rs715572, *TIMP3*rs9609643, *TIMP3*rs8136803, and *TIMP3*rs2234921 genotypes. Red blood cells were separated for measurement of lead and cadmium concentrations, and plasma was separated for measurement of selenium concentrations.

### 2.3. Arsenic, Cadmium, Lead, and Selenium Measurement

To ensure absence of arsenobetaine or arsenocholine (less toxic than inorganic arsenic and its methylated metabolites), high performance liquid chromatography was used to separate urinary arsenic species: arsenite (As^III^), arsenate (As^V^), and its metabolites, monomethylarsonic acid (MMA^V^) and dimethylarsinic acid (DMA^V^). Concentration of arsenic species was determined by hydride generator linked with atomic absorption spectrometry [25]. Plasma selenium and blood lead and cadmium concentrations were analyzed by inductively coupled plasma mass spectrometry [4]. If the experimental value was lower than the detection limit, the data analysis was carried out at the half-of-detection-limit concentration. The determination method, detection limit, reliability, and validity are shown in Supplementary Table S1. The sum of As^III^, As^V^, MMA^V^, and DMA^V^ concentrations was termed the total urinary arsenic concentration.

### 2.4. Determination of TIMP3 Gene Polymorphisms

Genomic DNA was extracted by digestion with proteinase K followed by phenol and chloroform. The Agena Bioscience MassARRAY System was used according to the manufacturer’s instructions to determine the *TIMP3*rs9619311, *TIMP3*rs11547635, *TIMP3*rs715572, *TIMP3*rs9609643, *TIMP3*rs8136803 and *TIMP3*rs2234921 genotypes.

### 2.5. Statistical Analysis

Continuous variables are presented as mean ± standard deviation, while categorical variables are presented as frequencies (percentages). The chi-square test was used to analyze the distribution of categorical variables among the groups of the subjects, and to test whether the *TIMP3* genotypes of the control group fitted a Hardy-Weinberg equilibrium. The Wilcoxon rank-sum test was conducted to compare the continuous variables between CKD cases and controls. Multiple logistic regression was used to evaluate the associations between *TIMP3* genotypes and CKD by estimating OR and 95% confidence interval (CI). All models were adjusted for confounders including age, sex, and educational level; consumption of alcohol, coffee, and tea; analgesic usage; and disease histories of diabetes and hypertension. All data were analyzed using SAS 9.4 software (SAS Institute, Cary, NC, USA). A two-sided *p*-value < 0.05 was considered significant.

## 3. Results

Table 1 shows sociodemographic characteristics, lifestyle, and disease history for CKD cases and controls. The percentage of educational level above high school was significantly higher in the controls than in CKD cases. There was no difference in the proportion of cigarette smoking between the two groups. The proportion of frequent or occasional consumption of alcohol, tea and coffee was significantly higher in controls than in CKD cases. The proportion of CKD patients who routinely used analgesics was higher than in controls. Significantly more CKD patients had hypertension and diabetes than controls.

Table 2 presents the association between *TIMP3* polymorphisms and CKD. The gene polymorphisms of *TIMP3*rs9619311, *TIMP3*rs11547635, *TIMP3*rs715572, *TIMP3*rs9609643, *TIMP3*rs8136803 and *TIMP3*rs2234921 were not associated with CKD.

Table 3 compares the total urinary arsenic, blood cadmium and lead, and plasma selenium concentrations between CKD and control groups. The total urinary arsenic and blood cadmium and lead concentrations were significantly higher, while plasma selenium levels were significantly lower in CKD cases than in controls.

We analyzed the association between *TIMP3* genotypes and CKD after stratifying for total urinary arsenic, blood cadmium and lead, and plasma selenium concentrations (Table 4). The median values of total urinary arsenic, blood cadmium and lead, and plasma selenium concentrations in the control group were used as cut-off points for stratification analysis. The OR of CKD in the *TIMP3*rs9609643 GA/AA genotype was significantly lower than that in GG genotype at high total urinary arsenic and blood lead concentrations, but not at low total urinary arsenic and blood lead concentrations. The OR of CKD in *TIMP3*rs8136803 GT/TT genotype was lower than that in GG genotype under low total urinary arsenic, and the OR of CKD in the *TIMP3*rs8136803GT/TT genotype was higher than in the GG genotype under low blood lead concentration. Thus, *TIMP3*rs9609643 and *TIMP3*rs8136803 may interact with total urinary arsenic or blood lead concentrations. Therefore, the combined effects of *TIMP3*rs9609643 and *TIMP3*rs8136803, and total urinary arsenic or and blood lead concentrations, on CKD were subsequently analyzed. However, under blood cadmium and plasma selenium stratification, no association between *TIMP3* polymorphisms and CKD was observed.

Pairwise analysis of combined effects of high total urinary arsenic or blood lead levels and *TIMP3* risk genotype is shown in Table 5. The OR of CKD was significantly increased in dose-response with no risk factor, one risk factor, or both risk factors. We observed 3.75-fold increased odds (95% CI 1.89–7.45) of CKD cases carrying the *TIMP3*rs9609643 GG genotype and high levels of blood lead (>37.44 μg/L) compared to controls. The *p*-value for the interaction term of *TIMP3*rs9609643 and blood lead concentration was 0.027, and it appeared that *TIMP3*rs9609643 had a multiplicative interaction with blood lead on CKD; however, the significance disappeared with the multivariate adjustment. Other interactions were not significant.

## 4. Discussion

We found the distributions of *TIMP3*rs9619311, *TIMP3*rs11547635, *TIMP3*rs715572, *TIMP3*rs9609643, *TIMP3*rs8136803 and *TIMP3*rs2234921 between CKD cases and controls did not differ. However, subjects with the *TIMP3*rs9609643 GA/AA genotype had a marginally or significantly lower OR of CKD than those with GG genotype at high total urinary arsenic or high blood lead concentration. The combined effect of *TIMP3*rs9609643 or *TIMP3*rs8136803 risk genotypes and high urinary total arsenic or high blood lead concentrations gradually increased the OR for CKD with increasing risk factors.

The pathogenesis of CKD is complex and may be caused by a combination of environmental, genetic, and other factors. According to our study, the *TIMP3*rs9609643 GA/AA genotype was significantly associated with lower risk of CKD compared with GG genotype at high total urinary arsenic or at high blood lead concentration. There are few studies on *TIMP3*rs9609643; of three studies in China, one explored the correlation between *TIMP3*rs9609643 and thoracic aortic dissection [26], one explored its relationship with osteoarthritis [27], and one explored the correlation with high myopia [28], but all had negative results. However, one study found that women with the *TIMP3*rs9609643 AA genotype were 60% less likely to develop breast cancer than women with the GG genotype (OR 0.4, 95% CI 0.2–1.0) [21].

However, we observed that the *TIMP3*rs8136803 GT/TT genotype decreased the risk of CKD at low total arsenic levels while increasing the risk of CKD at low blood lead concentrations, compared with the GG genotype. A current study found that the genotype and allele of *TIMP3*rs8136803 significantly differed between with and without primary open-angle glaucoma; the frequency of *TIMP3*rs8136803 genotype GG in primary open-angle glaucoma was higher than that in controls [29]. However, the *TIMP3*rs8136803 genotype was not associated with osteoarthritis [27] and one study indicated that women with the *TIMP3*rs8136803 TT genotype were five times more likely to develop breast cancer than those with the GG genotype (OR 5.1, 95% CI 1.1–24.3) [21]. In addition, breast cancer cases with *TIMP3*rs8136803 TT were almost four times more likely to have reduced disease-free survival and there was a trend toward reduced overall survival compared to the GG genotype [21].

A Chinese study found that *TIMP3*rs2234921 and *TIMP3*rs9619311 were associated with mixture plaque [19]. Another study pointed out that *TIMP3*rs9619311 is related to essential hypertension [20]. Studies found that *TIMP3*rs9619311 was associated with hepatocellular carcinoma [30] and colorectal cancer [31]. A recent study in Taiwan found that *TIMP3*rs9619311 was associated with survival in cervical cancer [32]. Additionally, *TIMP3*rs715572 was associated with colorectal cancer [22] and survival of adenocarcinoma of the gastroesophageal junction [33]. However, *TIMP3*rs9619311, *TIMP3*rs11547635, *TIMP3*rs715572 and *TIMP3*rs2234921 were not associated with CKD in our study, and there are also some studies with similar results to ours [34,35,36,37]. At present, there are few studies on the relationship between *TIMP3* genotype and disease and they have inconsistent results, so further investigation is needed. The functional relevance of these polymorphisms is also unclear. They may directly affect the expression or activity of *TIMP3*, or be markers for other functionally relevant variants, which requires further investigation.

Significantly gradually increased ORs for CKD with increasing risk factors (high total urinary arsenic concentration, high blood lead concentration, and *TIMP3*rs9609643 and *TIMP3*rs8136803 risk genotype) were observed in this study. This may be because exposure to arsenic, lead and cadmium induces oxidative stress and fibrosis, resulting in nephrotoxicity [6,7,8]. High concentrations of lead [16] or total urinary arsenic [38] reduced the expression of TIMP, leading to an imbalance in the MMPs/TIMPs ratio, favoring proteolytic enzyme activity and leading to generation of tissue abnormalities. In contrast, some studies found that long-term exposure to arsenic, possibly due to downregulation of *TIMP3*, and *TIMP3* deficiency may lead to oxidative stress [39], resulting in increased renal fibrosis [12,14]. However, the role of *TIMP3* in CKD remains unclear. Although the functional significance of the polymorphisms of *TIMP3*rs9609643 and *TIMP3*rs8136803 is unknown, some of the associations identified in our study support a possible role for these polymorphisms as, when combined with high total urinary arsenic or high blood lead concentration, they increased the OR of CKD.

This study had some limitations. The small number of homozygous individuals with rare alleles may have produced unstable OR estimates. Further studies with larger sample size are needed to improve the precision of point estimates when assessing *TIMP3* polymorphisms and environmental metals exposure in relation to CKD. The analysis of six *TIMP3* polymorphisms may not represent all the gene functions. Our study did not analyze gene polymorphisms regulating *TIMP3* expression. Further studies should be conducted to assess the function of *TIMP3* and its associated gene polymorphisms to determine their role in CKD development.

## 5. Conclusions

The risk of CKD related to high levels of blood lead or high levels of total urinary arsenic was modified by *TIMP3*rs9609643 GA/AA genotypes. High blood lead levels tended to interact with the *TIMP3*rs9609643 risk genotype to increase the risk of CKD. We recommend future studies of the levels of serum *TIMP3*, to determine the relevant mechanism regarding the relationships between CKD and *TIMP3* polymorphisms, and environmental metals exposure.

## Figures and Tables

**Table 1:** Sociodemographic characteristics, lifestyle, and disease histories for the CKD cases and controls.

Variables	CKD Cases (N = 214)	Controls (N = 423)	*p*
Age	64.99 ± 13.54	64.33 ± 12.58	0.486 ^a^
Gender			
Male	132 (61.68)	260 (61.47)	0.958 ^a^
Female	82 (38.32)	163 (38.53)	
Educational level			<0.0001 ^b^
Illiterate/elementary school	88 (41.12)	96 (22.70)	
Junior/senior high school	71 (33.18)	146 (34.52)	
College and above	55 (25.70)	181 (42.79)	
Cigarette smoking			0.678 ^b^
Non-smoker	157 (73.36)	307 (72.58)	
Former smoker	32 (14.95)	73 (17.26)	
Current smoker	35 (11.68)	43 (10.17)	
Alcohol consumption			<0.0001 ^b^
Never	177 (82.71)	270 (63.83)	
Frequently	33 (15.42)	65 (15.37)	
Occasional	4 (1.87)	88 (20.80)	
Coffee consumption			<0.0001 ^b^
Never	167 (78.04)	218 (51.05)	
Frequently	28 (13.08)	102 (24.11)	
Occasional	19 (8.88)	103 (24.35)	
Tea consumption			<0.0001 ^b^
Never	119 (55.61)	149 (34.89)	
Frequently	69 (32.24)	168 (39.72)	
Occasional	26 (12.15)	106 (25.06)	
Analgesic usage			<0.0001 ^b^
No/yes as-needed basis	188 (87.85)	404 (95.52)	
Yes, routinely	26 (12.15)	19 (4.48)	
Diabetes			<0.0001 ^b^
No	129 (60.28)	379 (89.60)	
Yes	85 (39.72)	44 (10.40)	
Hypertension			<0.0001 ^b^
No	92 (42.99)	296 (69.98)	
Yes	122 (57.01)	127 (30.02)	

Abbreviations: CKD, chronic kidney disease. Values are expressed as mean ± standard deviation or number (%) of cases and controls. ^a^ Wilcoxon rank-sum test. ^b^ χ^2^ test.

**Table 2:** Associations between *TIMP3* gene polymorphisms and CKD.

*TIMP3* Genotypes	CKD Cases	Controls	Age-Gender AdjustedORs (95% CI)	Multivariate AdjustedORs (95% CI) ^a^
rs11547635 C > T				
CC	96 (45.50)	195 (46.54)	1.00	1.00
CT	89 (42.18)	181 (43.20)	0.99 (0.70–1.42)	1.09 (0.72–1.67)
TT	25 (12.32)	43 (10.26)	1.23 (0.71–2.12)	1.12 (0.57–2.21)
rs2234921 A > G				
AA	181 (84.58)	348 (83.05)	1.00	1.00
AG	30 (14.02)	66 (15.75)	0.87 (0.55–1.39)	0.81 (0.47–1.42)
GG	3 (1.40)	5 (1.19)	1.12 (0.26–4.78)	0.92 (0.19–4.40)
rs715572 G > A				
GG	89 (41.59)	179 (42.82)	1.00	1.00
GA	104 (48.60)	189 (45.22)	1.11 (0.78–1.58)	1.16 (0.76–1.76)
AA	21 (9.81)	50 (11.96)	0.85 (0.48–1.50)	0.95 (0.48–1.90)
rs9609643 G > A				
GG	166 (77.57)	301 (71.67)	1.00	1.00
GA	46 (21.50)	109 (25.95)	0.77 (0.52–1.14)	0.74 (0.46–1.18)
AA	2 (0.93)	10 (2.38)	0.36 (0.08–1.66)	0.22 (0.03–1.65)
rs9619311 T > C				
TT	181 (84.98)	348 (82.86)	1.00	1.00
TC	29 (13.62)	67 (15.95)	0.83 (0.52–1.32)	0.80 (0.46–1.41)
CC	3 (1.41)	5 (1.19)	1.11 (0.26–4.74)	0.92 (0.19–4.44)
rs8136803 G > T				
GG	189 (88.32)	381 (90.07)	1.00	1.00
GT	23 (10.75)	37 (8.75)	1.25 (0.72–2.17)	0.88 (0.46–1.68)
TT	2 (0.93)	5 (1.18)	0.80 (0.15–4.15)	0.69 (0.12–4.12)

Abbreviations: CKD, chronic kidney disease; *TIMP3*, tissue inhibitor of metalloproteinase 3; OR, odds ratio; CI, confidence interval. Seven participants were missing for *TIMP3*rs11547635; four were missing for *TIMP3*rs2234921 and *TIMP3*rs9619311; three were missing for *TIMP3*rs9609643; and five were missing for *TIMP3*rs715572. ^a^ Adjusted for age, sex, educational level, analgesic usage, disease histories of diabetes and hypertension, and alcohol, coffee, and tea consumption. Multiple logistic regression models were used to calculate the association between *TIMP3* genotypes and CKD.

**Table 3:** Total urinary arsenic, blood cadmium and lead, and plasma selenium concentrations for CKD cases and controls.

	CKD Cases (N = 214)			Controls (N = 423)			
Variables	Median	First Quartile	Third Quartile	Median	First Quartile	Third Quartile	*p*
Total urinary arsenic(μg/g creatinine)	22.54	15.76	34.32	16.04	10.64	26.00	<0.0001
Blood cadmium (μg/L)	1.66	1.18	2.65	1.04	0.68	1.50	<0.0001
Blood lead (μg/dL)	63.65	41.44	88.15	37.44	25.56	52.64	<0.0001
Plasma selenium (μg/L)	185.68	147.80	223.45	217.85	182.60	253.15	<0.0001

*p*-value was tested by Wilcoxon rank-sum test.

**Table 4:** Associations between TIMP3 gene polymorphisms and CKD stratified by total urinary arsenic and blood lead concentrations.

	Total Urinary Arsenic > 16.04 μg/g Creatinine			Total Urinary Arsenic ≤ 16.04 μg/g Creatinine		
*TIMP3* Genotypes	CKD Cases/ Controls	Age-Sex Adjusted ORs (95% CI)	Multivariate Adjusted ORs (95% CI) ^a^	CKD Cases/ Controls	Age-Sex Adjusted ORs (95% CI)	Multivariate Adjusted ORs (95% CI) ^a^
rs9609643 G > A						
GG	124/143	1.00	1.00	42/158	1.00	1.00
GA/AA	34/67	0.55 (0.34–0.90) *	0.57 (0.31–1.05) ^+^	14/52	0.98 (0.49–1.95)	0.97 (0.45–2.12)
rs8136803 G > T						
GG	137/195	1.00	1.00	52/186	1.00	1.00
GT/TT	21/16	1.86 (0.94–3.71) ^+^	1.54 (0.67–3.54)	4/26	0.56 (0.19–1.70)	0.31 (0.09–1.09) ^+^
	Blood lead > 37.44 μg/dL			Blood lead ≤ 37.44 μg/dL		
rs9609643 G > A						
GG GA/AA	134/138 36/74	1.00 0.50 (0.31–0.79) **	1.00 0.52 (0.30–0.93) *	32/162 12/45	1.00 1.36 (0.65–2.85)	1.00 0.89 (0.37–2.17)
rs8136803 G > T						
GG	153/189	1.00	1.00	36/192	1.00	1.00
GT/TT	17/23	0.91 (0.47–1.76)	0.55 (0.24–1.26)	8/19	2.28 (0.92–5.63) ^+^	2.11 (0.74–1.51)

Abbreviations: *TIMP3*, tissue inhibitor of metalloproteinase 3; OR, odds ratio; CI, confidence interval. Seven participants were missing for *TIMP3*rs11547635; four were missing for *TIMP3*rs2234921 and *TIMP3*rs9619311; three were missing for *TIMP3*rs9609643; and five were missing for *TIMP3*rs715572. ^a^ Adjusted for age, sex, educational level, analgesic usage, disease histories of diabetes and hypertension, and alcohol, coffee, and tea consumption. ^+^ 0.05 < *p* < 0.1, ^*^ *p* < 0.05, ^**^ *p* < 0.01.

**Table 5:** Effects of interactions of *TIMP3* gene polymorphisms with total urinary arsenic and blood lead concentrations on CKD.

Metals	*TIMP3* Genotypes	CKD Cases/ Controls	Age-Gender Adjusted ORs (95% CI)	Multivariate Adjusted ORs (95% CI) ^a^
Total urinary arsenic (μg/g creatinine)	rs9609643 G > A			
≤16.04	GA/AA	14/52	1.00 ^&,^***	1.00 ^&,^***
≤16.04	GG	42/158	0.98 (0.50–1.95)	1.14 (0.53–2.43)
>16.04	GA/AA	34/67	1.91 (0.93–3.93) ^+^	1.87 (0.82–4.28)
>16.04	GG	124/143	3.29 (1.73–6.24) ***	3.10 (1.51–6.40) **
		Synergy index	2.57 (0.59–11.31)	2.08 (0.47–9.22)
		*p*_interaction_	0.197	0.761
Total urinary arsenic (μg/g creatinine)	rs8136803 G > T			
≤16.04	GT/TT	4/26	1.00 ^&,^***	1.00 ^&,^***
≤16.04	GG	52/186	1.80 (0.60–5.38)	3.21 (0.96–10.75) ^+^
>16.04	GG	137/195	4.58 (1.56–13.44) **	6.85 (2.08–10.75) **
>16.04	GT/TT	21/16	8.55 (2.48–29.48) ***	10.42 (2.60–41.66) ***
		Synergy index	1.72 (0.73–4.05)	1.17 (0.48–2.85)
		*p*_interaction_	0.979	0.797
Blood lead (μg/dL)	rs9609643 G > A			
≤37.44	GA/AA	12/45	1.00 ^&,^***	1.00 ^&,^***
≤37.44	GG	32/163	0.74 (0.35–1.55)	1.06 (0.44–2.52)
>37.44	GA/AA	36/74	1.86 (0.88–3.96)	3.13 (1.27–7.72) *
>37.44	GG	134/138	3.75 (1.89–7.45) ***	5.97 (2.60–13.67) ***
		Synergy index	4.58 (0.45–47.15)	2.27 (0.87–5.91)
		*p*_interaction_	0.027	0.137
Blood lead (μg/dL)	rs8136803 G > T			
≤37.44	GG	36/192	1.00 ^&,^***	1.00 ^&,^***
≤37.44	GT/TT	8/19	2.28 (0.93–5.61) ^+^	1.93 (0.69–5.42)
>37.44	GT/TT	17/23	4.02 (1.95–8.30) ***	3.32 (1.41–7.82) **
>37.44	GG	153/189	4.43 (2.91–6.67) ***	5.76 (3.44–9.66) ***
		Synergy index	0.80 (0.36–1.75)	1.46 (0.53–4.04)
		*p*_interaction_	0.647	0.563

Abbreviations: *TIMP3*, tissue inhibitor of metalloproteinase 3; OR, odds ratio; CI, confidence interval. ^&^ tested for linear trend. ^+^ 0.05 < *p* < 0.1, * *p* < 0.05, ** *p* < 0.01, *** *p* < 0.001. ^a^ Adjusted for age, sex, educational level, analgesic usage, disease histories of diabetes and hypertension, and alcohol, coffee, and tea consumption.

## Data Availability

The data presented in this study are available on request from the corresponding author. The data are not publicly available due to privacy or ethical restrictions.

## Supplementary Materials

The following supporting information can be downloaded at: https://www.mdpi.com/article/10.3390/ijerph20031886/s1, Table S1. The validity and reliability of urinary arsenic species, plasma selenium, and red blood cell lead and cadmium.
