# Supplementary material for: Chronic Kidney Disease: Combined Effects of Gene Polymorphisms of Tissue Inhibitors of Metalloproteinase 3, Total Urinary Arsenic, and Blood Lead Concentration
Source: Int J Environ Res Public Health. 2023 Jan 19;20(3):1886. doi: 10.3390/ijerph20031886 (PMC9914449; doi:10.3390/ijerph20031886)
Supplement: Supplementary file 1 [file ijerph-20-01886-s001.zip › ijerph-2084162-supplementary.pdf]

Supplementary Table

Supplementary Table S1. The validity and reliability of urinary arsenic species, plasma selenium, and red blood cell lead and cadmium.

Metals or metalloids	Method	Detection limit (µg/L)	Recovery rate	SRM	CV%
Plasma selenium	Inductively coupled plasma mass spectrometry	0.193		SRMs (Seronorm Trace Elements Whole Blood Label II (SERO AS, Norway) contained 112 ± 46 mg/L of selenium, in our system 118.7 ± 11.1 mg/L (n = 7)	9.8%
Red blood cell lead	Inductively coupled plasma mass spectrometry	0.32		SRMs (Seronorm Trace Elements Whole Blood L-2 (Lot 1103129)) certificate value 310.0 µg/L (range 186.0–434.0 µg/L), in our system 329.0 ± 17.0 µg/L	<10%
Red blood cell cadmium	Inductively coupled plasma mass spectrometry	0.07		SRMs (Seronorm Trace Elements Whole Blood L-2 (Lot 1103129) certificate value 5.8 µg/L (range: 5.4–6.2 µg/L), in our system 6.1 ± 0.5 µg/L	<10%
Arsenite (As^{III})	High-performance liquid chromatography-hydride generator-atomic absorption spectrometry	0.02	93.8–102.2%	SRM (National Institute of Standards and Technology (NIST, Gaithersburg, MD) 2670 certificate value 480 ± 100 µg/L inorganic arsenic, in our system 507 ± 17 µg/L (n = 4)	<10%
Arsenate (As^{V})		0.10			
Monomethylarsonic acid (MMA^{V})		0.07			
Dimethylarsinic acid (DMA^{V})		0.06			
